# Health among Retired Great Britain’s Olympic Athletes: A cross-sectional Study of Disease and Multimorbidity

**DOI:** 10.1186/s40798-025-00897-8

**Published:** 2025-08-07

**Authors:** Dale J. Cooper, Julius Sim

**Affiliations:** School of Allied Health Professions and Pharmacy, Keele University, Keele, Staffordshire, ST5 5BG UK

**Keywords:** Prevalence, Olympic Athletes, Multimorbidity, Chronic Diseases, Melanoma

## Abstract

**Background:**

Currently, there is a paucity of long-term health data for retired athletes. This study describes the prevalence of common morbidities and multimorbidity among retired Great Britain’s (GB) Olympic athletes, compared to a general population comparator group.

**Methods:**

A cross-sectional study of retired athletes was undertaken. The English Longitudinal Study of Ageing (ELSA) served as the reference population. Age- and sex-standardized morbidity ratios (SMRs) and odd ratios (ORs) determined where morbidity and multimorbidity prevalence amongst retired athletes exceeded or were inferior to those of the reference population.

**Results:**

Retired athletes (*n* = 493) were less likely (SMR < 1) than controls (*n* = 8024) to report diabetes (0.43, 99% CI 0.22, 0.74), stroke (0.39, 99% CI 0.12, 0.90), obesity (0.35, 99% CI 0.23, 0.50), asthma (0.29, 99% CI 0.12, 0.59), lung disease (0.29, 99% CI 0.06, 0.81), angina (0.18, 99% CI 0.05, 0.46), and eye disorders (0.06, 99% CI 0.01, 0.18). In addition, abnormal heart rhythm (0.45, 99% CI 0.40, 0.54) and osteoporosis (0.46, 99% CI 0.42, 0.51) were lower in female athletes. Retired athletes were more likely (SMR > 1) than controls to report melanoma or other skin cancer (5.64, 99% CI 2.80, 10.06) and osteoarthritis (1.44, 99% CI 1.18, 1.75). There were no differences detected in cancers of the breast, prostate, colon, bowel or bladder. Multimorbidity was less prevalent among retired athletes (OR 0.50, 99% CI 0.38, 0.67).

**Conclusions:**

Retired athletes had superior cardiovascular health and a lower risk of multimorbidity, but were more at risk of melanoma or other skin cancer and osteoarthritis.

**Supplementary Information:**

The online version contains supplementary material available at 10.1186/s40798-025-00897-8.

## Key Points

Retired British Olympic athletes were more at risk of melanoma or other skin cancer, and osteoarthritis, irrespective of sex, with skin cancer more common in those from outdoor sporting disciplines (sailing, rowing, athletics).

Compared to an age- and-sex matched general population comparator group, the risk of multimorbidity was lower in male and female retired Olympic athletes, and driven by superior cardiovascular health in those from endurance and power sporting disciplines.

Future athlete welfare and prevention strategies will need to adapt to different groups of athletes and to disease status in different body systems based on risk factors with the greatest impact.

**Supplementary Information:**

The online version contains supplementary material available at 10.1186/s40798-025-00897-8.

## Background

If we are to protect the long-term health of the athlete, we need to understand how engagement in sport impacts on health outcomes through the lifespan, and how disease interactions can influence morbidity status in the retired athlete. Currently, there is a paucity of knowledge regarding the long-term health outcomes of sports participation. Previous studies have examined the health of former athletes [1–9], typically focusing on individual disease status [1, 2, 6, 7, 9], cancer [10], and all-cause mortality [11]. However, studies that focus solely on mortality or specific disease status risk underreporting prevalent health conditions, leaving gaps in our knowledge of disease interactions, and how these may influence long-term health.

To our knowledge, no epidemiological studies have assessed the nature of multimorbidity in former male and female Olympic athletes. It is paramount for the protection of the athletes’ healthy lifespan that research identifies the common types of long-term conditions, the patterns of multimorbidity, and not just the number of diseases that coexist in a retired athlete. This approach will help to better understand the aetiology of disease and plan for optimal treatment. Understanding patterns of disease will help to identify ‘at-risk’ athletes and inform the design of screening pathways and interventions or preventive measures. The aim of this study was to describe the prevalence of common morbidities and of multimorbidity among retired Olympic athletes from Great Britain (GB), compared to a general population comparator group.

## Methods

### Study Design

This cross-sectional study involved distributing a letter by post or email giving retired athletes the opportunity to complete and return a postal questionnaire or complete an online web-based version. The questionnaire was distributed through the British Olympic Association (BOA) Athletes’ Commission to individuals who had represented Great Britain at the Summer and/or Winter Olympic Games. Responses from retired athletes were included if they were no longer training to qualify for or compete at an upcoming Olympic Games. A detailed description of the recruitment of retired athletes is described elsewhere [4]. Data for the comparator group were extracted from the English Longitudinal Study of Ageing (ELSA), a prospective, population-based cohort study of community-dwelling male and female adults living in England, aged 50 years or older [12]. ELSA participants are invited to follow-up interviews at two-yearly time points, known as waves, and a medical examination every four years. For the present study, wave 6 was selected as the cross-sectional comparator group due to the availability of anthropometric data and to coincide with the timing of recruitment of retired athletes.

### Research Ethics Approval

Ethics approval for the recruitment of retired athletes was granted by the University of Nottingham Research Ethics Committee (Ref: K13022014). Retired athletes received detailed information at the start of the questionnaire, detailing how data would be handled to ensure confidentiality and anonymity. All procedures involving retired athletes were in accordance with the ethical standards of the university institution review board. The ELSA dataset was used as a representative population-based survey comparator group [13]. Ethics approval for ELSA was previously provided by the NHS Research Ethics Committees under the National Research and Ethics Service (NRES). Anonymized ELSA data for the present study were provided by the UK Data Service.

### Data Collection and Management

The questionnaire collected detailed information from retired athletes, including demographics, history of medical conditions, and sporting career [4]. Baseline questions captured self-reported information on age (years), sex, height (cm), weight (kg), ethnicity, and occupation. Body mass index (BMI) was calculated by dividing the weight in kilograms by the square of height in meters (kg/m^2^). BMI was coded according to: underweight (< 18.50), normal weight (18.50 to 24.99), overweight (pre-obese) (*≥* 25.00 to < 30.00), and obese (*≥* 30.00). Physical and mental health conditions were included based on their availability within the ELSA dataset, clinical relevance, and disease burden [14]. The historical diagnoses were captured at ‘any time’ to ensure information on disease status was integrated at the index date of participant inclusion. To avoid spurious associations, any morbidity with a prevalence of less than 1% in retired athletes was excluded [15]. In total, 16 conditions were catalogued singly and grouped according to six system-specific chapters, following the International Classification of Diseases, Tenth Revision (ICD 10). Each condition was coded as a dichotomous variable, where presence = 1 and absence = 0. Data recorded as a ‘don’t know’ or ‘refusal’ responses were recorded as missing and excluded from the analysis. Socioeconomic status was classified according to the UK National Statistics Socio-Economic Classification self-coding method based on occupational data and categorized into the following main categories: (i) routine/manual, (ii) intermediate occupations, and (iii) professional occupations. All participants were classified according to the occupation they had practised for the longest period. Sports were categorized in line with other research, according to the acute physiological response (heart rate and blood pressure) and long-term impact on cardiac output and remodelling [16, 17]. Sports were also categorized according to: (i) endurance sports, defined as any sport requiring more than 10 min of prolonged and intensive high-dynamic exercise (long-distance runners, cyclists); (ii) power sports of less than 45 s, mostly anaerobic (weight lifters, sprinters); (iii) mixed sports, defined by alternating phases of dynamic and/or static work and recovery (football, hockey); and (iv) skilled sports, dependent on technical or bodily skill (shooting, archery).

### Definition

Multimorbidity was defined as the co-existence of two or more long-term diseases without considering an index disease [14], whereas complex multimorbidity was defined as the co-occurrence of three or more long-term diseases affecting three or more body systems without considering an index disease [18].

### Power Calculation

A power calculation was performed in GPower V.3.1.9.2, using a logistic regression model with a post-hoc power analysis. In the sample of 493 exposed (athletes) and 8024 unexposed (comparison group), assuming all exposures could at least be dichotomized, this study had at least 81% power to detect an odds ratio of 1.23 or greater at 1% two-tailed significance, based on a 50.6% prevalence rate of multimorbidity in the general population compactor group.

### Statistical Analysis

Descriptive statistics are presented for the retired athletes and the ELSA comparison group. Frequencies and percentages are presented for categorical variables, and for numerical variables data are presented with the mean and standard deviation. Between-group differences in demographics were compared using the unpaired *t*-test for continuous variables, the *X*^2^ test for binary variables, and the *X*^2^ test for trend for ordinal variables. The crude prevalence of morbidity was calculated as the number of participants with the condition divided by the respective study population. Age- and sex-standardized morbidity ratios (SMRs) were estimated to demonstrate where morbidity prevalence amongst retired athletes exceeded or was lower than that of the standard population. This method of adjustment used 10-year age increments in accordance with previous literature [9]. Age-, sex-, and occupation-adjusted odds ratios (OR) with 99% confidence intervals (CIs) were calculated through logistic regression to estimate the odds of multimorbidity. Preliminary analysis was undertaken to ensure there was no violation of the assumption of multicollinearity, as indicated by *r≥*0.7 and a variance inflation factor > 10. The analysis was restricted to those aged 50 years and older because conditions such as osteoarthritis and multiple long-term conditions are rare in the general population. Statistical significance was accepted at *p* *≤* 0.01, with corresponding 99% CIs, and analyses were performed using SPSS v 29.0 (SPSS Inc., Chicago, IL, USA), apart from the SMRs, which were calculated using the method of indirect standardization via an internet-based application (http://fingertips.phe.org.uk).

## Results

**Fig. 1:**
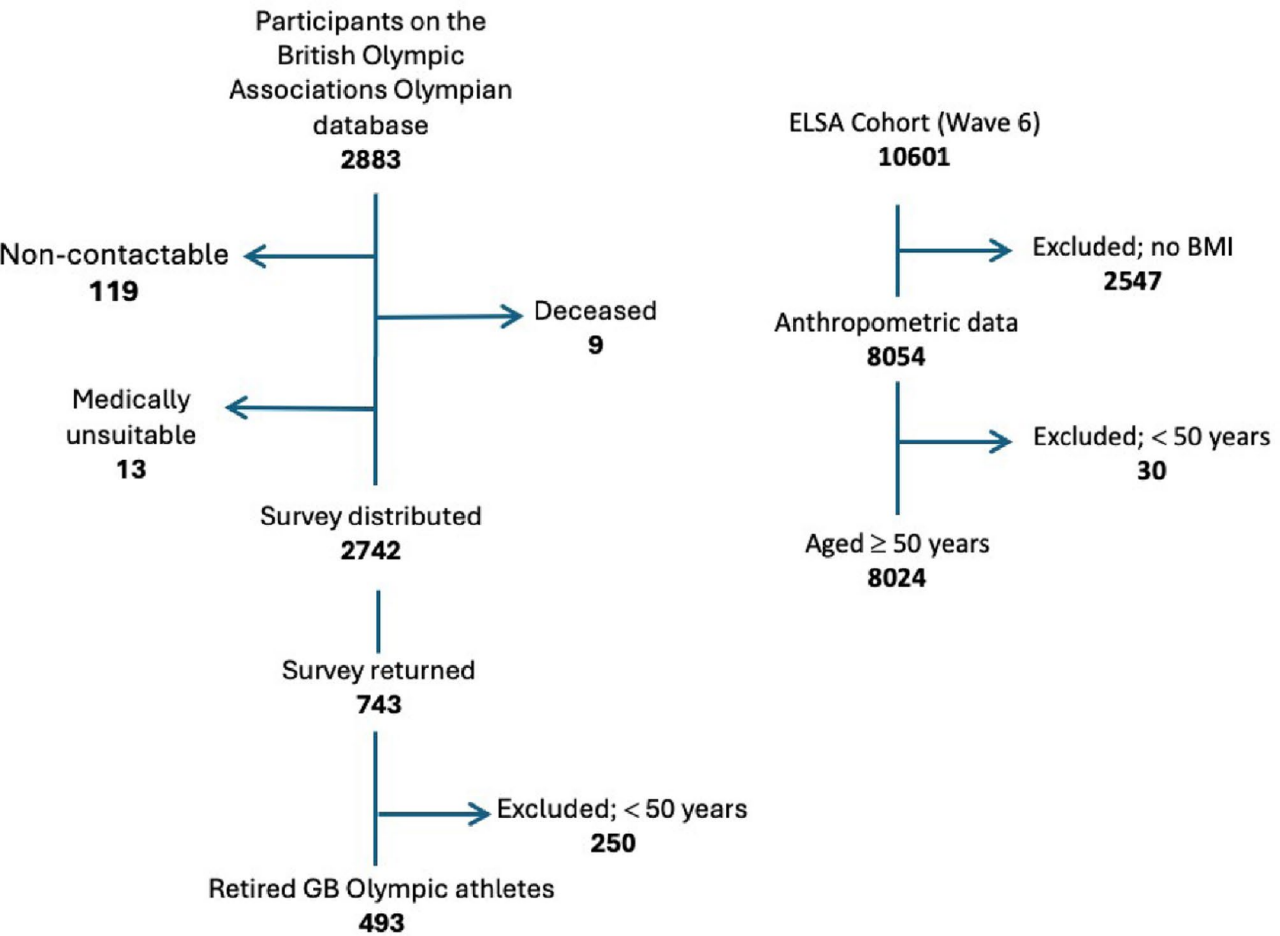
Flow chart (left) describes the number of retired athletes included in this study from the British Olympic Association database. The chart describes those who could not be contacted, the number of surveys distributed, the number of surveys returned, and the number of surveys included in the analysis. The flow chart (right) describes the number of participants from ELSA (wave 6) included in this study, and those excluded because they were under 50 years of age or had no recorded BMI data

The questionnaire was distributed to 2742 athletes registered on the BOA Olympian database. Of the 743 who replied (27.1% response rate), 493 were retired from their Olympic careers and had data for the analysis. Of the 10,601 participants in Wave 6 of ELSA, 8024 were included in the analysis on the basis of having arthrometric data recorded (Fig. 1).

Table 1 reports the characteristics of the participants. The mean age of retired athletes (67.7 years) was comparable to that of the controls (67.0 years). There were fewer female retired athletes (35.7%) compared to the proportion of females in the reference population (55.2%). The mean BMI among retired athletes (25.0 kg/m^2^) was 3.3 kg/m^2^ lower than that of the comparison group (28.3 kg/m^2^). A detailed breakdown of the sports that retired athletes previously competed in is provided in the supplemental material A. In summary, retired athletes had competed in 10 sports at the Winter Olympics (*n* = 50) and in 22 sports at the Summer Olympics (*n* = 443).

**Table 1:** Characteristics of the retired GB Olympic athletes and ELSA cohorts

	All, *n* = 8517	Retired GB Olympic Athletes, *n* = 493	ELSA Population, *n* = 8024	*P* value
Age (years), mean (SD)	67.1 (9.7)	67.7 (11.0)	67.0 (9.6)	0.309 (*t*)
Age group, n (%)				
50–59	1993 (23.4)	144 (29.2)	1849 (23.0)	**< 0.001** (*X*^2^_t_)
60–69	3249 (38.1)	138 (28.0)	3111 (38.8)	
70–79	2259 (26.5)	121 (24.5)	2138 (26.6)	
80+	1016 (11.9)	90 (18.3)	926 (11.5)	
Height (cm), mean (SD)	166.2 (10.2)	174.4 (15.0)	165.7 (9.5)	**< 0.001** (*t*)
Weight (kg), mean (SD)	77.8 (16.5)	77.0 (15.7)	77.8 (16.6)	0.295 (*t*)
BMI (Kg/m^2^), mean (SD)	28.1 (5.2)	25.0 (4.0)	28.3 (5.3)	**< 0.001** (*t*)
Female, n (%)	4602 (54.0)	176 (35.7)	4426 (55.2)	**< 0.001** (*X*^2^)
Ethnicity (white), n (%)	8259 (97.0)	480 (97.4)	7779 (96.9)	0.698 (*X*^2^)
Any current disease, n (%)	6515 (76.5)	326 (66.1)	6189 (77.1)	**0.001** (*X*^2^)
Any current medication, n (%)	4238 (49.8)	276 (56.0)	3962 (49.4)	**0.002** (*X*^2^)
Polypharmacy (≥ 5 medicines), n (%)	85 (1.0)	32 (6.5)	53 (0.7)	**0.001** (*X*^2^)

The *p* values represent comparison between retired GB Olympic athletes and the standard population controls, using unpaired *t*-test (*t*) for continuous variables, the chi-square test (*X*^2^) for binary variables, and the chi-square test for trend (*X*^2^_t_) for ordinal categories. Statistically significant differences are highlighted in bold

### Prevalence of Morbidity in Retired Athletes Versus Controls

Table 2 shows the prevalence of the conditions analysed. The most prevalent morbidities amongst retired athletes were osteoarthritis (36.1%, 99% CI 30.4%, 41.7%), hypertension (27.4%, 99% CI 22.2%, 32.7%), cancer (13.6%, 99% CI 9.5%, 17.6%), and obesity (9.8%, 99% CI 6.3%, 13.3%). The most prevalent morbidities amongst the controls were hypertension (34.0%, 99% CI 32.7%, 35.4%), obesity (29.9%, 99% CI 28.6%, 31.2%), osteoarthritis (28.3%, 99% CI 27.0%, 29.7%), and eye disorders (16.9%, 99% CI 15.8%, 18.0%). The most common types of cancer in controls were prostate (2.6%, 99% CI 1.9%, 3.3% [males]), breast (2.1%, 99% CI 1.5%, 2.6% [females]), melanoma or other skin cancer (0.6%, 99% CI 0.4%, 0.8%), and colon, bowel or rectum (0.5%, 99% CI 0.3%, 0.8%). The most prevalent types of cancers among retired athletes were melanoma or other skin cancer (3.7%, 99% CI 1.5%, 5.9%), prostate (3.2%, 99% CI 0.6%, 5.8% [males]), breast (2.3%, 99% CI 0.0%, 5.3% [females]), and colon, bowel or rectum (1.6%, 99% CI 0.2%, 3.1%) (supplemental material B).

### Standardized Morbidity Ratios (SMRs)

Table 2 also shows the age- and sex-adjusted SMRs for the long-term conditions. An SMR with an upper 99% confidence limit below 1.0 indicates a statistically significant lower prevalence in retired athletes, while an SMR with a lower 99% confidence limit greater than 1.0 indicates a statistically significant higher prevalence in retired athletes, compared to the standard population. Retired athletes were more likely to report cancer (2.14, 99% CI 1.52, 2.91), and osteoarthritis (1.44, 99% CI 1.18, 1.75), but less likely to report diabetes mellitus (0.43, 99% CI 0.22, 0.74), stroke (0.39, 99% CI 0.12, 0.90), obesity (0.35, 99% CI 0.23, 0.50), asthma (0.29, 99% CI 0.12, 0.59), lung disease (0.29, 99% CI 0.06, 0.81), angina pectoris (0.18, 99% CI 0.05, 0.46), and visual problems (0.06, 99% CI 0.01, 0.18). In addition, the prevalence of hypertension, rheumatoid arthritis, abnormal heart rhythm, osteoporosis, and myocardial infarction were lower in retired athletes (SMR < 1), but the SMRs did not reach statistical significance. Melanoma or other skin cancers were more common in retired athletes than in controls (5.64, 99% CI 2.80, 10.06). However, no significant differences in SMR were observed between athletes and controls for cancers of the breast, prostate, colon, bowel or bladder.

**Table 2:** Prevalence and standardized morbidity ratios for each condition in retired athletes versus the reference population

Morbidity		% ELSA (99% CI), Total (*n* = 8024) Males (*n* = 3598) Females (*n* = 4426)	% Olympians (99% CI), Total (*n* = 493) Males (*n* = 317) Females (*n* = 176)	SMR, adjusted for age and sex (99% CI)
Circulatory disorders:				
Hypertension	T	34.0 (32.7, 35.4)	27.4 (22.2, 32.7)	0.81 (0.64, 1.00)
	M	35.5 (33.4, 37.5)	30.7 (23.9, 37.4)	0.82 (0.62, 1.07)
	F	32.9 (31.0, 34.7)	21.5 (13.3, 29.7)	0.76 (0.48, 1.15)
Angina pectoris	T	6.2 (5.5, 6.9)	1.2 (0.2, 2.5)	**0.18 (0.05**,** 0.46)**
	M	7.1 (6.0, 8.2)	1.9 (0.1, 3.9)	**0.23 (0.06**,** 0.60)**
	F	5.6 (4.7, 6.4)	–	–
Myocardial infarction	T	4.8 (4.2, 5.5)	3.9 (1.6, 6.2)	0.62 (0.31, 1.11)
	M	7.0 (5.9, 8.1)	4.6 (1.5, 7.8)	0.57 (0.25, 1.10)
	F	3.1 (2.4, 3.8)	2.4 (0.0, 5.6)	0.90 (0.80, 1.08)
Abnormal heart rhythm	T	6.3 (5.6, 7.0)	4.1 (1.8, 6.4)	0.61 (0.31, 1.05)
	M	6.9 (5.8, 8.0)	5.1 (1.9, 8.3)	0.66 (0.31, 1.22)
	F	5.9 (4.9, 6.8)	2.3 (0.0, 5.3)	**0.45 (0.40**,** 0.54)**
Stroke	T	3.6 (3.0, 4.1)	1.6 (0.2, 3.1)	**0.39 (0.12**,** 0.90)**
	M	3.9 (3.1, 4.8)	1.9 (0.1, 3.9)	**0.38 (0.10**,** 0.98)**
	F	3.3 (2.6, 4.0)	1.2 (0.0, 3.3)	**0.42 (0.34**,** 0.59)**
Respiratory:				
Asthma	T	9.4 (8.6, 10.3)	2.5 (0.6, 4.3)	**0.29 (0.12**,** 0.59)**
	M	7.9 (6.8, 9.1)	2.6 (0.2, 4.9)	**0.33 (0.11**,** 0.76)**
	F	10.6 (9.4, 11.8)	2.3 (0.0, 5.3)	**0.24 (0.04**,** 0.76)**
Chronic lung disease*	T	3.5 (3.0, 4.1)	1.0 (0.0, 2.2)	**0.29 (0.06**,** 0.81)**
	M	3.8 (3.0, 4.7)	1.3 (0.0, 2.9)	0.32 (0.05, 1.00)
	F	3.3 (2.6, 4.0)	0.6 (0.0, 2.1)	**0.21 (0.13**,** 0.36)**
Endocrine, nutritional & metabolic:				
Diabetes mellitus	T	9.3 (8.5, 10.2)	4.1 (1.8, 6.5)	**0.43 (0.22**,** 0.74)**
	M	10.9 (9.6, 12.2)	5.5 (2.1, 8.8)	**0.49 (0.24**,** 0.89)**
	F	8.1 (7.0, 9.1)	1.7 (0.0, 4.3)	**0.25 (0.03**,** 0.90)**
Obesity	T	29.9 (28.6, 31.2)	9.8 (6.3, 13.3)	**0.35 (0.23**,** 0.50)**
	M	28.0 (26.1, 30.0)	10.5 (6.0, 15.0)	**0.40 (0.24**,** 0.61)**
	F	31.4 (29.6, 33.2)	8.6 (3.1, 14.2)	**0.28 (0.27**,** 0.29)**
Mental health & behavioural:				
Anxiety	T	5.9 (5.2, 6.5)	5.9 (3.1, 8.7)	1.08 (0.63, 1.73)
	M	5.0 (4.0, 5.9)	6.2 (2.6, 9.7)	1.37 (0.70, 2.41)
	F	6.6 (5.6, 7.5)	5.4 (0.8, 9.9)	0.75 (0.26, 1.66)
Depression	T	7.1 (6.4, 7.9)	6.7 (3.8, 9.7)	1.02 (0.61, 1.58)
	M	5.8 (4.8, 6.8)	7.8 (3.8, 11.8)	1.49 (0.82, 2.47)
	F	8.2 (7.1, 9.3)	4.8 (0.5, 9.1)	0.52 (0.17, 1.21)
Neoplasm:				
Cancer (any)	T	5.9 (5.2, 6.6)	13.6 (9.5, 17.6)	**2.14 (1.52**,** 2.91)**
	M	6.4 (5.5, 7.6)	14.0 (8.9, 19.1)	**2.03 (1.33**,** 2.96)**
	F	5.4 (4.6, 6.3)	12.7 (6.1, 19.3)	**2.39 (1.28**,** 4.04)**
Melanoma or other skin cancer	T	0.6 (0.4, 0.8)	3.7 (1.5, 5.9)	**5.64 (2.80**,** 10.06)**
	M	0.7 (0.4, 1.1)	3.2 (0.6, 5.8)	**4.41 (1.63**,** 9.45)**
	F	0.5 (0.2, 0.8)	4.6 (0.4, 8.8)	**8.66 (2.78**,** 20.11)**
Prostate	T	–	–	–
	M	2.6 (1.9, 3.3)	3.2 (0.6, 5.8)	1.07 (0.39, 2.29)
	F	–	–	–
Colon, bowel or bladder	T	0.5 (0.3, 0.8)	1.6 (0.2, 3.1)	2.42 (0.78, 5.62)
	M	0.7 (0.4, 1.1)	1.6 (0.0, 3.4)	2.23 (0.48, 6.32)
	F	0.4 (0.2, 0.6)	1.7 (0.0, 4.3)	2.82 (0.32, 10.32)
Breast	T	–	–	–
	M	–	–	–
	F	2.1 (1.5, 2.6)	2.3 (0.0, 5.3)	1.20 (0.20, 3.79)
Eye disorders:				
Glaucoma +/- cataracts	T	16.9 (15.8, 18.0)	1.1 (0.0, 2.3)	**0.06 (0.01**,** 0.18)**
	M	14.5 (12.9, 16.0)	1.3 (0.0, 3.0)	**0.08 (0.01**,** 0.24)**
	F	18.8 (17.3, 20.4)	0.6 (0.0, 2.1)	**0.04 (0.03**,** 0.07)**
Musculoskeletal & connective tissue:				
Osteoporosis	T	6.7 (6.0, 7.5)	2.9 (0.9, 4.9)	0.65 (0.29, 1.25)
	M	1.9 (1.3, 2.5)	2.3 (0.1, 4.5)	1.13 (0.33, 2.77)
	F	10.7 (9.5, 11.9)	4.1 (0.1, 8.1)	**0.46 (0.42**,** 0.51)**
Osteoarthritis (any joint)	T	28.3 (27.0, 29.7)	36.1 (30.4, 41.7)	**1.44 (1.18**,** 1.75)**
	M	21.5 (19.7, 23.3)	34.9 (27.9, 41.9)	**1.58 (1.22**,** 2.02)**
	F	33.9 (32.1, 35.8)	38.2 (28.5, 47.8)	**1.26 (1.25**,** 1.29)**
Rheumatoid arthritis	T	6.7 (6.0, 7.5)	4.1 (1.8, 6.5)	0.64 (0.33, 1.11)
	M	5.5 (4.5, 6.5)	5.4 (2.1, 8.8)	0.91 (0.44, 1.65)
	F	7.7 (6.7, 8.8)	1.7 (0.0, 4.3)	**0.24 (CI 0.03**,** 0.88)**

Prevalence (%) and standardized morbidity ratios (SMRs), with 99% confidence intervals (CIs), adjusted for age and sex for each condition in retired GB Olympic athletes and in the ELSA (reference) population. For each condition, estimates are shown for the total (T) and for male (M) and female (F) retired Olympic athletes and controls. Statistically significant SMRs are highlighted in bold. *Chronic bronchitis or emphysema

After stratifying by sex, age-adjusted SMRs indicated that female retired athletes, compared to female controls, were less likely to report eye problems (0.04, 99% CI 0.03, 0.07), lung disease (0.21, 99% CI 0.13, 0.36), asthma (0.24, 99% CI 0.04, 0.76), rheumatoid arthritis (0.24, 99% CI 0.03, 0.88), diabetes (0.25, 99% CI 0.03, 0.90), obesity (0.28, 99% CI 0.27, 0.29), stroke (0.42, 99% CI 0.34, 0.59), abnormal heart rhythm (0.45, 99% CI 0.40, 0.54), and osteoporosis (0.46, 99% CI 0.42, 0.51). Conversely, cancer (2.39, 99% CI 1.28, 4.04) and osteoarthritis (1.26, 99% CI 1.25, 1.29) were higher in female retired athletes compared to female controls. In comparison, male retired athletes, compared to male controls, were less likely to report eye problems (0.08, 99% CI 0.01, 0.24), angina (0.23, 99% CI 0.06, 0.60), asthma (0.33, 99% CI 0.11, 0.76), stroke (0.38, 99% CI 0.10, 0.98), obesity (0.40, 99% CI 0.24, 0.61), and diabetes (0.49, 99% CI 0.24, 0.89). Male retired athletes were also more likely to report cancer (2.03, 99% CI 1.33, 2.96), and osteoarthritis (1.58, 99% CI 1.22, 2.02).

### Multimorbidity in Retired Athletes Versus Controls

**Fig. 2:**
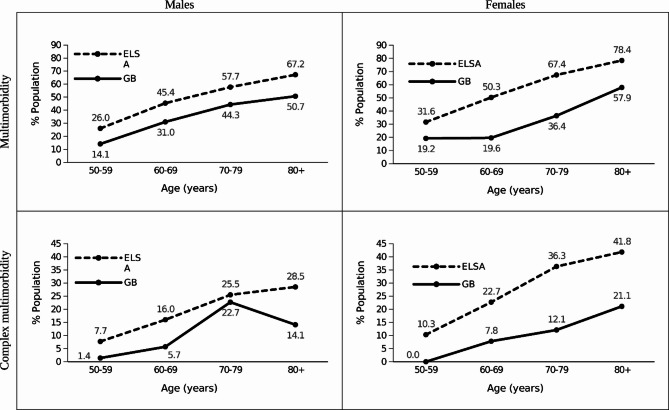
Crude prevalence (%) of multimorbidity by sex, 10-year age group, and multimorbidity type for retired GB Olympic athletes compared to the standard population

Multimorbidity was more prevalent with increasing age both in male and female retired athletes and in male and female controls (Fig. 2). Complex multimorbidity became more prevalent with older age and peaked in the 80 + age group for male and female controls, and in female retired athletes, but peaked in the 70–79 age group, before declining with age, in male retired athletes. Both types of multimorbidity were more prevalent in each age group among male and female controls, compared to male and female retired athletes.

Overall, the odds of multimorbidity (adjusted for age, sex, and occupation) were lower for retired athletes compared with the standard population (prevalence 32.3% vs. 50.6%; OR 0.50, 99% CI 0.38, 0.67; *p* < 0.001). After stratification by sex, the odds (adjusted for age and occupation) of multimorbidity were lower for male retired athletes, compared with male controls (prevalence 35.3% vs. 46.8%; OR 0.58, 99% CI 0.40, 0.82; *p* < 0.001), and lower in female retired athletes, compared with female controls (prevalence 26.7% vs. 53.8%; OR 0.40, 99% CI 0.24, 0.66; *p* < 0.001). The odds of complex multimorbidity (adjusted for age, sex, and occupation) were lower for retired athletes, compared with the standard population (prevalence 9.7% vs. 22.3%; OR 0.43, 99% CI 0.28, 0.68; *p* < 0.001). After stratification by sex, the odds (adjusted for age and occupation) of complex multimorbidity were lower in male retired athletes, compared with male controls (prevalence 11.4% vs. 18.1%; OR 0.58, 99% CI 0.34, 0.97; *p* = 0.006), and lower in female retired athletes, compared with female controls (prevalence 6.8% vs. 25.7%; OR 0.24, 99% CI 0.10, 0.59; *p* < 0.001).

**Fig. 3:**
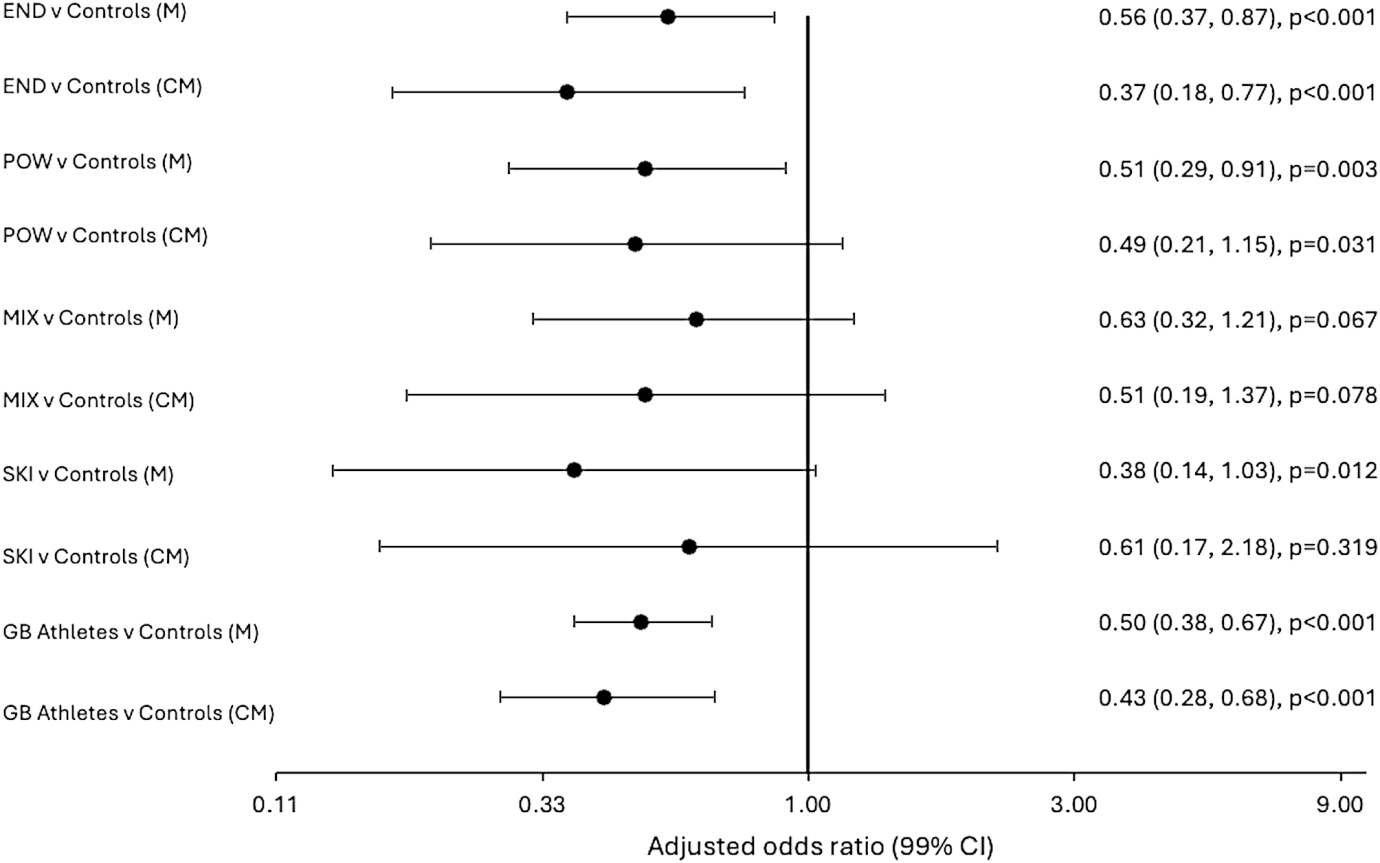
Forest plot showing adjusted (age, sex, and occupation) odds ratios of multimorbidity (M) and complex multimorbidity (CM) among retired GB Olympic athletes (all, and 4 sporting disciplines) versus controls. Sporting disciplines: endurance (END), power (POW), mixed (MIX), and skilled disciplines (SKI). Multimorbidity is defined by two or more long-term conditions. Complex multimorbidity is defined by three or more different body systems affected by disease

After stratifying by sport versus controls, multimorbidity (adjusted for age, sex, and occupation) was less prevalent among athletes from endurance (prevalence 32.4% vs. 50.6%; OR 0.56, 99% CI 0.37, 0.87; *p* < 0.001) and power disciplines (prevalence 33.3% vs. 50.6%; OR 0.51, 99% CI 0.29, 0.91; *p* = 0.003). This pattern did not extend to those from mixed (prevalence 39.8% vs. 50.6%; OR 0.63, 99% CI 0.32, 1.21; *p* = 0.067) and skilled disciplines (prevalence 27.9% vs. 50.6%; OR 0.38, 99% CI 0.14, 1.03; *p* = 0.012) (Fig. 3). Similarly, compared to controls, complex multimorbidity (adjusted for age, sex, and occupation) was less prevalent among athletes from endurance disciplines (prevalence 7.4% vs. 22.3%; OR 0.37, 99% CI 0.18, 0.77; *p* < 0.001). However, after adjustment, no significant differences were observed among retired athletes from mixed (prevalence 12.0% vs. 22.3%; OR 0.51, 99% CI 0.19, 1.37; *p* = 0.078) or skilled disciplines (prevalence 14.0% vs. 22.3%; OR 0.61, 99% CI 0.17, 2.18; *p* = 0.319). For power athletes, complex multimorbidity did not reach the significance threshold (prevalence 12.3% vs. 22.3%; OR 0.49; 99% CI 0.21, 1.15; *p* = 0.031).

## Discussion

This study described the prevalence of common morbidities and multimorbidity among retired Great Britain’s Olympic athletes, compared to a general (reference) population comparator group. The main findings are that: (i) retired athletes were more likely to report melanoma or other skin cancer, and osteoarthritis; (ii) retired athletes were less likely to report eye disorders, angina, asthma, lung disease, obesity, stroke, and diabetes; (iii) female retired athletes were also less likely to report rheumatoid arthritis, abnormal heart rhythm, and osteoporosis; and (iv) the prevalence of multimorbidity was lower among retired athletes, with superior cardiovascular health observed among individuals from endurance and power disciplines.

### Cancer

Regular moderate exercise is widely accepted to protect against several types of cancer, including bladder, breast, colon, endometrial, and kidney cancers. This protection may be attributed to lower levels of systemic inflammation, insulin-like growth factor, estrogen, and pro-inflammatory leptin, as well as increased levels of the anti-inflammatory hormone adiponectin [10, 19]. Former athletes are reported to benefit from participating in aerobic exercise through a lower cancer mortality risk [11]. Other studies have confirmed that this lower cancer risk is attributable to a specific lower risk of smoking-related cancers, such as lung and renal cancers [10, 20]. The view that exercise protects all athletes against cancer may be challenged by participation in outdoor sports increasing the exposure to harmful ultraviolet radiation and consequent risk of skin cancers (melanoma, squamous cell carcinoma and basal cell carcinoma) [21, 22]. Exercise does not protect against non-melanoma skin cancer [23], and chronic systematic inflammation linked to overtraining may induce immunosuppression, which can increase the risk of skin cancers, including melanoma [23, 24]. Sweating induced by exercise increases the photosensitivity of the skin, thereby increasing the risk of sunburn from ultraviolet radiation [25]. Our study observed 7.7% more cases of cancer in retired athletes compared to age- and sex-matched population controls. The most prevalent cancer type among retired athletes (melanoma or other skin cancer) was present in those from outdoor sporting disciplines (sailing, rowing, athletics). There remains the possibility that the increased diagnosis of melanoma among retired Olympic athletes may be partially influenced by differences in healthcare access, potentially leading to earlier detection and more effective treatment. Such differences could help explain the higher observed frequency of melanoma and other skin cancers in athletes in our study. However, our cross-sectional data do not allow us to determine whether the elevated prevalence in athletes is due to disparities in healthcare access or reflects the longer lifespans previously reported in this population [11], which may increase their lifetime risk. Alternatively, the elevated prevalence may be attributed to environment factors, such as prolonged outdoor training leading to greater exposure to ultraviolet radiation.

### Osteoarthritis

The present study detected 7.8% more cases of physician-diagnosed osteoarthritis in retired athletes compared to the reference population. This finding is consistent with published studies that report increased rates of osteoarthritis in former athletes [7, 8, 26]. However, former studies have predominantly focused on the hip and knee, whereas our findings relate to osteoarthritis at any joint. Previous studies have confirmed that career-related injury is high among retired athletes and is associated with osteoarthritis in later life [4]. Joint injury is an established risk factor for the onset of osteoarthritis in the general population. In young adults, a sixfold increased risk of future knee osteoarthritis has been found following cruciate ligament injury, meniscal tear, and intra-articular fracture [27]. Our findings illustrate that proactive management of osteoarthritis is needed among Olympic athletes and particularly among those competing in power and team sport disciplines. Injury prevention programmes are needed to minimize the long-term health risks associated with joint injury.

### Osteoporosis

Low bone mass and osteoporosis occur in more than 158 million adults worldwide, disproportionately affecting more women than men [28]. Regular physical activity is an established intervention to protect against osteoporosis by improving bone mineral density and peak bone mass in both sexes, when undertaken before and/or after growth [29, 30]. Our study found female retired athletes were at a lower risk of osteoporosis compared to female controls, but this did not extend to male retired athletes compared to male controls. Osteoporosis was a rare outcome in both male cohorts, and there may not be sufficient statistical power to detect differences. Previous studies have found female elite athletes have higher bone mineral density than non-athletic controls, and athletes in high-impact sports have higher bone mineral density compared to those in moderate-impact and low-impact sports [31]. Our study found no association between competing in high- and low-impact sports and the risk of osteoporosis. The retired athletes’ training regimens frequently comprised both weight-bearing and non-weight-bearing activities in high- and low-impact sports.

### Cardiovascular Disease

Regular physical activity is known to reduce the risk of cardiovascular disease. The scientific evidence supporting the long-term cardiovascular benefits in retired athletes is limited, and notably there is a lack of data in retired female athletes to establish their cardiovascular risk profile. Our study found retired athletes had a lower risk of symptoms of coronary heart disease (angina). Furthermore, retired athletes had a lower risk of symptoms associated with cerebrovascular disease including stroke and, specifically in female athletes, cardiac arrythmias. Our study also observed retired athletes had a lower prevalence of known risk factors for cardiovascular disease, obesity and diabetes. These finding are consistent with previous literature that found members of the Italian team selected as potential competitors at the Olympics had a lower prevalence of risk factors for cardiovascular disease [32]. Our study supports regular exercise as a key strategy for prevention of cardiovascular events. Other established risk factors for cardiovascular disease – including tobacco use, low-quality diet, household air pollution, abdominal obesity, and non-high-density lipoprotein (HDL) cholesterol – remain unexplored in retired athletes [33].

### Other Morbidities

Morbidities that did not differ significantly between retired athletes and controls included anxiety, depression, rheumatoid arthritis, hypertension, and cancers of the breast, prostate, colon, bowel or bladder. Although the SMRs did not reach the significance threshold, the prevalences of anxiety and depression were lower in female retired athletes compared to female controls, and higher in male retired athletes compared to male controls. Rheumatoid arthritis was less prevalent in female retired athletes, compared to female controls. Eye disorders were significantly less prevalent in retired athletes, adding further support to a growing body of literature that suggests that higher physical activity levels are associated with better eye health [34].

### Multimorbidity

Individuals with multiple long-term conditions are more likely to have poorer health and a lower quality of life [35, 36]. Our study found that multimorbidity (2 or more conditions) increased with age and was more prevalent in controls than in retired athletes, irrespective of sex. However, multimorbidity was less prevalent in endurance and power athletes, but not in mixed and skilled disciplines, compared to controls. Complex multimorbidity measured the number of body systems affected (3 or more body systems). Our study found complex multimorbidity was more prevalent in controls than in retired athletes, irrespective of sex, and was less prevalent in endurance athletes, but not in mixed, power, and skilled disciplines. It remains unclear whether participation in endurance events or the specific morphology of athletes in these disciplines is responsible for their lower risk of multimorbidity. Overall, these findings imply that retired athletes suffer fewer chronic conditions, which are typically associated with increased healthcare utilization and a need for coordinated care among healthcare professions [35].

### Limitations

This study is not without limitations. Firstly, there is a possibility of response bias, and retired athletes who have poorer health and/or multimorbidity may have had a greater propensity to partake in this study. To mitigate the risk of recruitment bias, we made strenuous efforts to send the questionnaire to all retired GB athletes living in 30 different countries. Secondly, this is a cross-sectional study and may be subject to recall bias. However, the risk of recall bias is arguably offset by reporting on long-term health conditions that are present and are therefore more likely to be recalled. Thirdly, long-term diseases among athletes were excluded if they were not reported within the comparator group. However, none of the unreported conditions reached the reporting threshold of 1% prevalence and were therefore excluded to avoid spurious associations. Additionally, there was consistency between self-reported outcomes across both cohorts and comparability of the question phraseology between our survey and ELSA; however, our definition of multimorbidity relied on the frequency of conditions and is highly variable depending on the number of conditions included in the study. Nonetheless, we used the most common definition previously used in epidemiological studies, and managing more than one health condition translates into a larger treatment burden [37, 38]. A large number of associations were reported, but we used a conservative cutoff for statistical significance (*p*≤ 0.01) to limit the rate of Type 1 errors. Finally, although adjustments were made for age, sex and occupation, there may be residual confounding by other variables for which analyses were not adjusted.

## Conclusion

This study reports early and new important work on the long-term health of retired Olympic athletes. Compared to an age- and sex-matched general population comparator group, retired athletes were more likely to report cancer and osteoarthritis, irrespective of sex. The greater risk of cancer resulted from melanoma or other skin cancer in those from outdoor sporting disciplines (sailing, rowing, athletics). The risk of multimorbidity was lower in retired athletes and driven by superior cardiovascular health status observed in endurance and power disciplines. This study supports the implementation of targeted athlete welfare and prevention strategies to lower the risk of skin cancer and osteoarthritis. Future prevention strategies will need to adapt to different groups of athletes and to disease status in different body systems based on risk factors with the greatest impact. Finally, a global observational study is needed to determine individual cancer rates among retired athletes from other National Olympic Committees.

## Electronic Supplementary Material

Below is the link to the electronic supplementary material.

Supplementary Material 1

## Data Availability

An anonymized summary of the GB Olympian data set generated and analysed during the current study may be available from the corresponding author on reasonable request. The individual-level comparator dataset analysed during the current study is available in the English Longitudinal Study of Ageing, https://www.elsa-proje ct.ac.uk/accessing-elsa-data.

## Abbreviations

SMR: Standardized morbidity ratio
BOA: British Olympic Association
GB: Great Britain
ELSA: English Longitudinal Study of Ageing
BMI: Body mass index
OR: Odds ratio
CI: Confidence interval
