# Supplementary material for: Health among Retired Great Britain’s Olympic Athletes: A cross-sectional Study of Disease and Multimorbidity
Source: Sports Med Open. 2025 Aug 7;11:93. doi: 10.1186/s40798-025-00897-8 (PMC12332171; doi:10.1186/s40798-025-00897-8)
Supplement: Supplementary file 1 — Supplementary Material 1 [file 40798_2025_897_MOESM1_ESM.docx]

**APPENDIX A**

Supplemental A: Figure. A breakdown of the sports that retired GB Olympic athletes (n=493) had competed in at the Summer and Winter Olympic Games. Retired GB athletes had competed in 10 sports at the Winter Olympics (n=50) and 22 sports at the Summer Olympics (n=443).

Supplemental B: Table. Frequency (percentage) of cancer types in retired athletes versus the reference population.

|  | ELSA (n=8024) | Retired athletes (n=487) |
| --- | --- | --- |
| Breast [females only] | 91 (2.1) | 4 (2.3) |
| Colon, bowel or rectum | 44 (0.5) | 8 (1.6) |
| Leukaemia | 4 (0.05) | – |
| Lymphoma | 12 (0.1) | – |
| Lung | 8 (0.1) | 2 (0.4) |
| Melanoma or other skin cancer | 49 (0.6) | 18 (3.7) |
| Prostate [males only] | 93 (2.6) | 10 (3.2) |
| Other | 170 (2.1) | 24 (4.9) |
| Total | 471 (5.9) | 66 (13.6) |
